# Development of an Anticipatory Triage-Ranking Algorithm Using Dynamic Simulation of the Expected Time Course of Patients With Trauma: Modeling and Simulation Study

**DOI:** 10.2196/44042

**Published:** 2023-06-15

**Authors:** Manuel Sigle, Leon Berliner, Erich Richter, Mart van Iersel, Eleonora Gorgati, Ives Hubloue, Maximilian Bamberg, Christian Grasshoff, Peter Rosenberger, Robert Wunderlich

**Affiliations:** 1 University Department of Anesthesiology and Intensive Care Medicine University Hospital Tübingen Eberhard Karls University Tübingen Germany; 2 University Department of Cardiology and Angiology University Hospital Tübingen Eberhard Karls University Tübingen Germany; 3 University Department of Pediatrics and Adolescent Medicine Ulm University Medical Center Ulm Germany; 4 Interactive Simulation Emergency Exercise support limited company Wemmel Belgium; 5 Emergency Department Universitair Ziekenhuis Brussel Brussel Belgium; 6 Research Group on Emergency and Disaster Medicine Vrije Universiteit Brussel Brussel Belgium; 7 German Society for Disaster Medicine (Deutsche Gesellschaft für Katastrophenmedizin) Kirchseeon Germany

**Keywords:** novel triage algorithm, patient with trauma, dynamic patient simulation, mathematic model, artificial patient database, semisupervised generation of patients with artificial trauma, high-dimensional analysis of patient database, Germany, algorithm, trauma, proof-of-concept, model, emergency, triage, simulation, urgency, urgent, severity, rank, vital sign

## Abstract

**Background:**

In cases of terrorism, disasters, or mass casualty incidents, far-reaching life-and-death decisions about prioritizing patients are currently made using triage algorithms that focus solely on the patient’s current health status rather than their prognosis, thus leaving a fatal gap of patients who are under- or overtriaged.

**Objective:**

The aim of this proof-of-concept study is to demonstrate a novel approach for triage that no longer classifies patients into triage categories but ranks their urgency according to the anticipated survival time without intervention. Using this approach, we aim to improve the prioritization of casualties by respecting individual injury patterns and vital signs, survival likelihoods, and the availability of rescue resources.

**Methods:**

We designed a mathematical model that allows dynamic simulation of the time course of a patient’s vital parameters, depending on individual baseline vital signs and injury severity. The 2 variables were integrated using the well-established Revised Trauma Score (RTS) and the New Injury Severity Score (NISS). An artificial patient database of unique patients with trauma (N=82,277) was then generated and used for analysis of the time course modeling and triage classification. Comparative performance analysis of different triage algorithms was performed. In addition, we applied a sophisticated, state-of-the-art clustering method using the Gower distance to visualize patient cohorts at risk for mistriage.

**Results:**

The proposed triage algorithm realistically modeled the time course of a patient’s life, depending on injury severity and current vital parameters. Different casualties were ranked by their anticipated time course, reflecting their priority for treatment. Regarding the identification of patients at risk for mistriage, the model outperformed the Simple Triage And Rapid Treatment’s triage algorithm but also exclusive stratification by the RTS or the NISS. Multidimensional analysis separated patients with similar patterns of injuries and vital parameters into clusters with different triage classifications. In this large-scale analysis, our algorithm confirmed the previously mentioned conclusions during simulation and descriptive analysis and underlined the significance of this novel approach to triage.

**Conclusions:**

The findings of this study suggest the feasibility and relevance of our model, which is unique in terms of its ranking system, prognosis outline, and time course anticipation. The proposed triage-ranking algorithm could offer an innovative triage method with a wide range of applications in prehospital, disaster, and emergency medicine, as well as simulation and research.

## Introduction

### Background

Worldwide, the risk of terrorism, natural disasters, and conflicts is taking on new shapes and sizes with every passing year [1]. With over 2 million fatalities in the past 2 decades and many more wounded and traumatized people [1,2], the global burden of disease has gained another protagonist.

Disasters have never waited their turn, and at the moment of sudden and overwhelming confrontation with a disaster or mass casualty incident, health care professionals have to make far-reaching decisions that make a life-or-death difference for patients. To ensure the ability to work under this pressure, to standardize these decisions, and to initiate concrete courses of action, triage algorithms have been developed. These algorithms enable the quick, simple, and robust prioritization of casualties with varying degrees of injury. By their nature, disaster and mass casualty events require rapid identification and management of life-threatening injuries following a systematic and standardized approach [3]. Accurate triage ensures that limited medical resources are directed toward achieving the greatest-possible positive impact for the largest number of people [2,4]. When patients are overtriaged (ie, when noncritically injured people are supplied with immediate care), resources are quickly depleted and treatment delay could occur for others. In contrast, undertriage (ie, when individuals with life-threatening problems are mistakenly underprioritized) can lead to adverse consequences or death [5]. It is therefore evident that the accuracy of triage decisions critically affects casualty prognosis and the overall success of the medical response to a disaster [5-7].

### Limitations in Current Triage

Currently, the established triage algorithms have 3 critical limitations:

- The triage category assignment depends solely on the patient’s current vital signs. In the most common triage algorithm (ie, Simple Triage And Rapid Treatment [START]) [8], neither the severity of the injury nor the expected time course of the individual patient is considered.
- Resource limitations are not considered. When resources are scarce, the policy of “worst first” (ie, patients with the worst injuries/vital parameters need to be treated before less severely injured patients) yields poor results [9]. In such cases, triage should prioritize patients in less critical conditions but with greater chances of survival, thus achieving an improvement in the expected number of lives saved [9].
- Triage resulting in 4-5 categories does not represent a final solution for prioritizing patients, as there could be a wide range of survival probabilities within the same triage category.

### Study Aims

Since START was developed, consensus emerged that triage should be more sophisticated (eg, by incorporating resource limitations and patient priority determination) [5,9]. In this proof-of-concept study, we demonstrate an entirely novel approach for triage to address the enumerated issues. We aimed at developing an approach that no longer classifies patients into triage categories but instead ranks them according to a mathematical model comprising current vital parameters and injury severity. Both variables were integrated using the well-established Revised Trauma Score (RTS) [10] and the New Injury Severity Score (NISS) [11], offering a priori high levels of feasibility, comparability, and validity. We specifically sought to establish a model that fulfills both the requirement of simple and rapid data acquisition during prehospital assessment and the high sensitivity of triage classification using a sophisticated mathematical model.

## Methods

### Study Design

This work is a proof-of-concept study that aims to underline the need for a novel triage algorithm and demonstrate the feasibility of applying a unique triage classification model by using a realistic large-scale patient database. The study does not claim external validity, as a real patient cohort was not used. Instead, this study illustrates a valid form of hypothesis and model testing using an artificial patient database. Due to the unpredictable nature of disasters, victims cannot consent to treatment or study enrollment, and ethical inadequacy limits data generation. For this reason, we decided to generate this artificial patient database, as described later.

**Figure 1:**
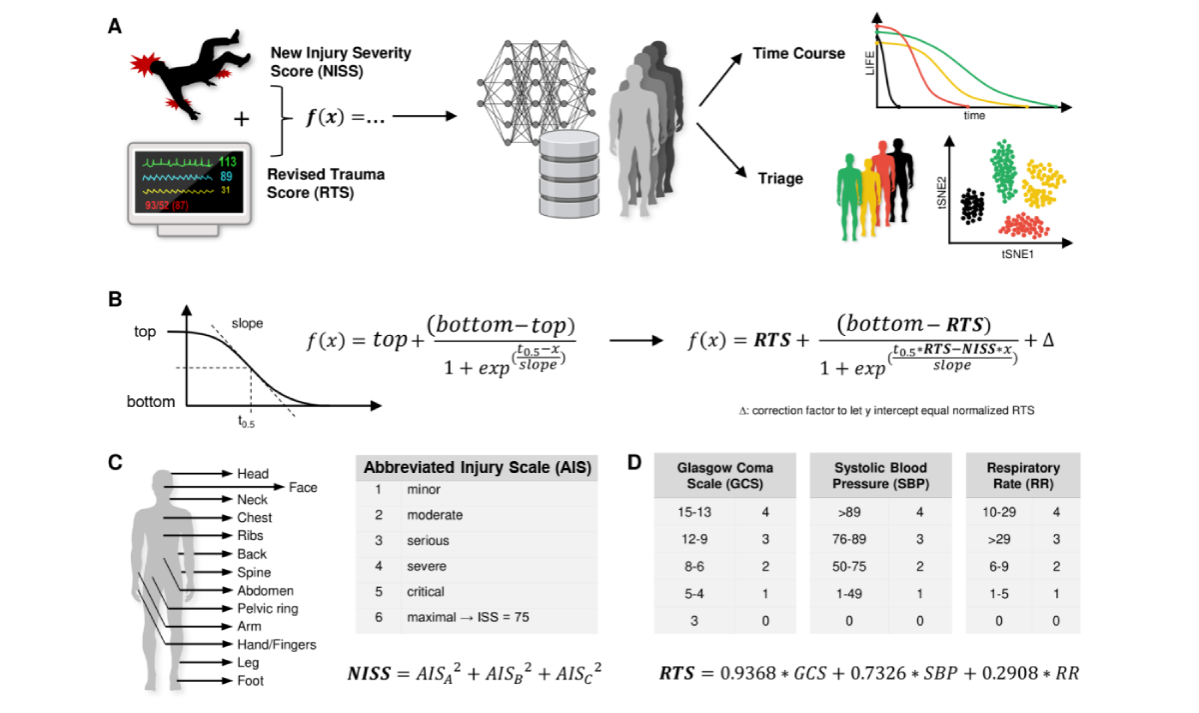
Conception of the study and the model. (A) Workflow overview. We started with a combination of vital signs and injury severity to fit a mathematical model, generated an artificial patient database, and then performed tests and simulations for time course modeling, triage prioritization, and multidimensional analysis of these patients. (B) Mathematical backbone of the LIFE triage model, described by the Boltzmann sigmoidal function and its adaptation to integrate vital signs by the RTS and injury severity by NISS. (C, D) Parameters considered in NISS (C) and the RTS (D) and their calculation. AIS: Abbreviated Injury Scale; GCS: Glasgow Coma Scale; NISS: New Injury Severity Score; RR: Respiratory Rate; RTS: Revised Trauma Score; SBP: Systolic Blood Pressure; t-SNE: t-distributed stochastic neighbor embedding.

The overall design of this study is illustrated in Figure 1A: we aimed to generate a mathematical model that simulates a patient’s “life percentage” (hereafter LIFE percentage) based on the current vital signs (using the RTS) and injury severity (using the NISS). This model enabled us to predict how long a patient would survive if they did not receive any treatment. Depending on this “survivalTimeWithoutTreatment,” we could rank the urgency of treatment for a patient. To test this model on as many patients as possible, we consecutively generated a large-scale patient database, where each patient has a unique injury pattern and realistic corresponding vital parameters. We then ran our model with patients from the database to verify the temporal trend of parameters and the triage-ranking capacities, and we compared our model with established triage algorithms.

### Establishment of the LIFE Triage Model

The sigmoid semilogarithmic Boltzmann equation was used as the mathematical backbone of the LIFE triage model (Figure 1B). This function has been widely used in biomedical research to describe diverse biological situations of increase or decrease [12,13]. Due to ethical inadequacy, there is no evidence on the time course of the LIFE percentage or vital parameters of patients with trauma who do not receive any treatment. Hence, modeling the time course of patients is based on empirical assumptions. In trauma research, several experimental studies illustrate the shape of the Boltzmann function in situations of biological decline (eg, the influence of blood loss on continuously monitored vital signs) [14,15]. The course of the basic Boltzmann function replicates the expected physiological behavior of the life of a patient with trauma; that is, after the traumatic event, there is a compensation period, where the central nervous system counterbalances blood loss and pain perception by vegetative adaptations [15-18], followed by a decompensation period of a linear decline in the LIFE percentage and a prolonged fatal ending. We modified the Boltzmann function by implementing the RTS and NISS as variables (Multimedia Appendix 1).

### Semisupervised Generation of Patients With Trauma

**Figure 2:**
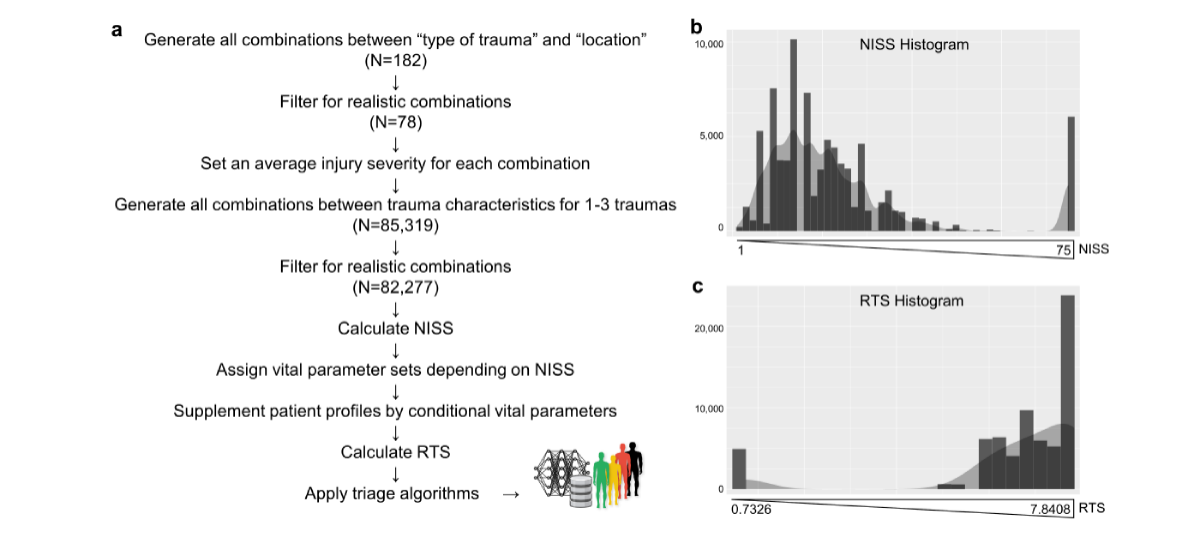
Semisupervised generation of a large-scale artificial patient database. (A) Scheme of the generation of the patient database. (B) Frequency distribution of the NISS over all 82,277 patients in the artificial database. Most patients show minor injury severity. (C) Frequency distribution of the RTS among all patients. At the lowest injury severity, most patients have good vital signs. NISS: New Injury Severity Score; RTS: Revised Trauma Score.

The workflow to generate the artificial patient database is shown in Figure 2A and is elaborated in detail in Multimedia Appendix 1. Briefly, we predefined 14 different types of traumas and 13 body locations and created all combinations between these 2 variables (Multimedia Appendix 1, Figure S3). Only realistic combinations were selected, and injury severity was set according to the Abbreviated Injury Scale (AIS) (Figure 1C). Next, all combinations of 1-3 traumas were iterated through the previously defined combinations. Patients with anatomically impossible combinations of 3 similar injuries in 2 extremities or 2 injuries in the azygos body regions were removed. Next, the NISS was calculated based on the AIS provided before. Depending on the NISS, we assigned vital parameter sets specified in Multimedia Appendix 1, Figure S4. Single vital parameters were randomly chosen from the specified range defined there. The basic vital parameters specified in the sets were consequently supplemented by depending vital parameters, as shown in Multimedia Appendix 1, Figure S3. As concrete examples, the parameter of low blood pressure increases the capillary refill time of that specific patient, and radial pulse palpability is not possible in the patient if the systolic blood pressure is less than 80 mmHg [19]. Intentionally, the assigned vital parameters were kept within realistic ranges (Multimedia Appendix 1, Figure S4) rather than providing concrete values, as we would also expect high heterogeneity among patients in a realistic mass casualty incident, due to preexisting conditions, age, general health, injury pattern, etc. Moreover, not all patients are treated at the same time, due to limited resources or complicated access to the patients. As the histograms in Figures 2B and 2C highlight, most of the patients (N=82,277) from the database suffer from injuries, with NISS=14-20 (range 1-75), and have, respectively, high RTS values of mostly 6-7.8408 (range 0.7326-7.8408).

Next, the RTS was calculated using the aforementioned vital parameters. Conclusively, various triage algorithms could be applied to the data set (Multimedia Appendix 1).

### High-Dimensional Analysis of the Patient Database

During the assessment of patients with trauma, numerous parameters are measured to evaluate the urgency of care for a patient. We aimed to objectify this evaluation by considering all the data collected during the primary assessment. We used our previously generated patient database, which includes information about the vital signs of patients and the pattern and severity of the injuries. As patients with similar features should have similar triage categories, a multidimensional analysis of the features of these patients would lead to a cluster. Clustering allows for a better understanding of the whole patient data set generated before and the performance of the different triage algorithms on all patients. We used the Gower distance [20] to measure the similarity and dissimilarity in the mixed data on patient features. Depending on the variable scale type, a particular Gower metric was used and scaled to fall between 0 and 1. Subsequently, Gower scaled data were clustered using the partitioning around medoids algorithm provided by the R package *cluster*. This algorithm is more robust to noise than the widely known k-means algorithm and has the benefit of calculating a characteristic, average patient for each cluster, which is a real patient from the data set [21,22]. To visualize the multidimensional data set in 2D space, we applied t-distributed stochastic neighbor embedding (t-SNE) [23] as the dimension reduction technique and used the Gower distance as a custom distance metric.

Clustering analysis of the patient data set was performed with the aim of separating patients with similar injury patterns and vital signs into distinct clusters. The variables used for the multidimensional analysis are shown in Figure S7 (Multimedia Appendix 1). To find the optimal cluster count, the silhouette plot was evaluated, and the number of clusters was chosen as k=6 (Multimedia Appendix 1, Figure S7).

## Results

### Modeling the Time Course of Patients With Trauma

Current triage models categorize patients into immediate (“red”), delayed (“yellow”), minimal/minor (“green”), and deceased/expectant (“black”) groups. This categorization is based on the evaluation of vital parameters or injury severity [2,8,24-26]. However, variability within a triage group is not considered. Patients with initially stable vital signs can rapidly deteriorate due to high injury severity. Additionally, patients with poorer vital signs at the initial assessment but low injury severity could survive longer than expected. In fact, current triage algorithms do not consider the dynamic component of trauma over time. To overcome these limitations, we established the LIFE triage model, which continuously ranks patients for treatment priority, predicting the individual course in the absence of treatment. The mathematical backbone of this model is a modified Boltzmann function with the RTS and NISS implemented. Both variables influence the course of the LIFE percentage curve, as outlined in Multimedia Appendix 1.

**Figure 3:**
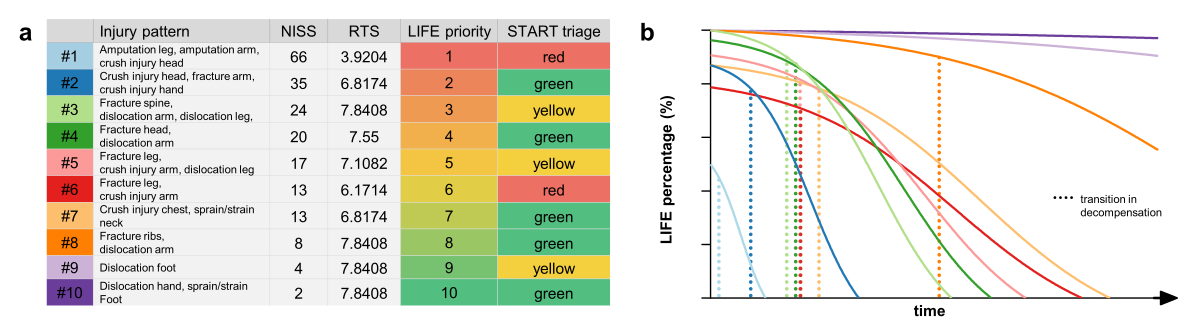
Modeling of the time course of vital signs. Simple simulation of a mass casualty incident involving 10 people injured in a multiple-vehicle collision. (A) LIFE triage correctly ranked patients according to their vital signs and injury severity and outperformed START in accuracy and prognosis prediction. (B) Time course model of patients from the simulation. Dotted vertical lines represent the transition into the decompensation stage. NISS: New Injury Severity Score; RTS: Revised Trauma Score; START: Simple Triage And Rapid Treatment.

To demonstrate the benefits and feasibility of our novel approach to triage, we ran a simple simulation of a mass casualty incident. In this fictive scenario, there were 10 people injured in a multiple-vehicle collision, all with different traumatic injury patterns (Figure 3A). The patients were selected from our database and are listed in Multimedia Appendix 1, Figure S6. According to the calculated “survivalTimeWithoutTreatment,” patients were ranked for their priority (LIFE priority). The calculated time course of the LIFE percentage is plotted in the corresponding color in Figure 3B (right). START identified n=2 (20%) immediate patients in the “red” triage category, n=3 (30%) in the “yellow” triage category, and n=5 (50%) in the “green” triage category. Unexpectedly, patient #2 was ranked as second priority, while START assigned a “green” triage category to this patient. The high injury severity (NISS=35) but currently stable vital parameters (RTS=6.8174) indicated that START mistriaged the patient as “green” because they were still able to walk. In contrast, patient #6 was triaged as “red” but had lower priority. This is due to the minor injury severity and stable vital parameters. Interestingly, triage by our algorithm could also discriminate between the priority of patient #6 versus patient #7, as both patients had the same NISS, but patient #7 had better vital signs. Additionally, at least in this scenario, it should be noted that the LIFE priority ranking was not only driven by the NISS, which shows the expected order of injury severity among the patients, but also driven by RTS. As Figure 3B visualizes, patients can also have good vital signs at the initial assessment but can rapidly deteriorate due to high injury severity. Hence, our LIFE model set a higher priority for this patient in comparison to patients with better vital signs.

As a result, our anticipatory triage-ranking algorithm successfully identified patients at risk and outperformed START in terms of accuracy in this scenario. We also concluded that stratification by the RTS or the NISS alone would not have been sufficient to replicate the prioritization calculated by our model.

### Different Triage Algorithms Classify Similar Patients Differently

**Figure 4:**
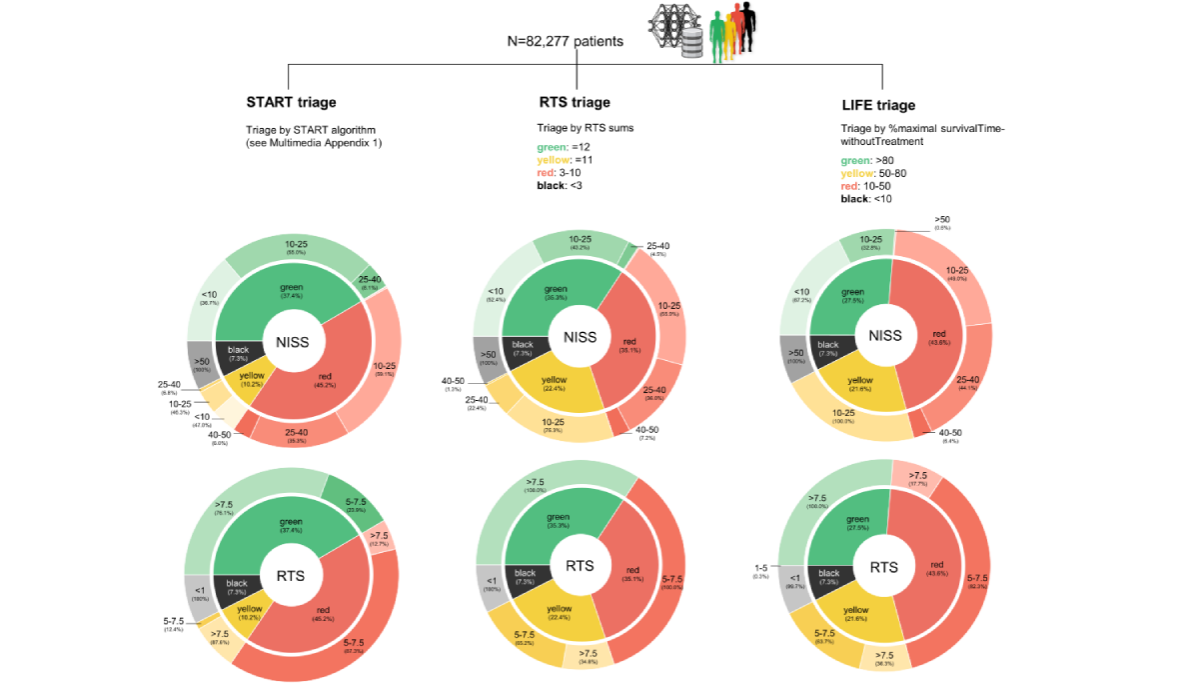
Different triage classifications by different triage algorithms. Comparative performance analysis of different triage algorithms (START, RTS, and LIFE triage) on all patients from the artificial patient database. The multilevel pie charts show the size of the triage category (inner ring) and the underlying NISS (top row) or RTS (bottom row). Interestingly, START assigned several patients to the “green” triage category despite having an NISS of 25-40 or an RTS of 5-7.5. In contrast, the LIFE and RTS triage models classified more patients as “yellow.” NISS: New Injury Severity Score; RTS: Revised Trauma Score; START: Simple Triage And Rapid Treatment.

To study a larger cohort of patients, all individuals from the artificial patient database were analyzed for their assignment to triage categories according to START’s triage algorithm, the RTS triage algorithm [24], and our LIFE triage algorithm (Figure 4A). Minor computational modifications to the algorithms are outlined in Multimedia Appendix 1. Since triage is based on the ranking of expected survival times in our proposed LIFE triage model, we set triage categories using ranges of the maximal “survivalTimeWithoutTreatment” (specified in Figure 4A, top right). For the visualization, we used a multilevel donut chart, where the inner ring outlines the size of the triage category and the outer ring outlines the corresponding NISS (top) or RTS (bottom) of the specific triage category.

The overall triage category assignment indicates that the number of patients in the “black” triage category was equal (n=6007, 7.3%), whereas patients in the “yellow” triage category displayed the highest variance (START triage: n=8393, 10.2%; RTS triage: n=18,430, 22.4%; LIFE triage: n=17,772, 21.6%). When looking at patients in the “green” triage category classified by START, 2493 (8%) patients with NISS=25-40 were most likely undertriaged and mistakenly assigned to the minor category. RTS triage misclassified 1315 (4.5%) patients in the green category, whereas LIFE triage did not assign any patient with NISS>25 to the “green” triage category. Interestingly, all patients who were assigned to the “yellow” triage category by LIFE triage had NISS=10-25, underlining a strong influence of injury severity on the triage algorithm.

When analyzing the distribution of the RTS among the triage algorithms, it is striking that although START substantially relies on vital parameters for triage classification, patients with an RTS of 5-7.5 (n=7354, 23.9%, in the “green” triage category) were also assigned to this group. In contrast, all patients assigned to the “green” triage category by LIFE triage had RTS>7.5. It should also be noted that there was a discrepancy between the RTS and RTS triage, which is because the RTS weights its factors differently, whereas RTS triage is performed by summing up the RTS values (see Figure 1D and Multimedia Appendix 1).

### Unsupervised Clustering Algorithm Replicates Clusters of Specific Triage Groups and Patient Features

Triage is based on the categorization of patients with similar clinical characteristics. These characteristics can be vital signs; the type, location, and severity of injuries; the ability to walk; the presence of critical bleeding, etc. Especially when numerous variables need to be assessed, it becomes difficult for humans to consider all factors in the same way. Therefore, machine learning methods have been developed that can compute hundreds of variables, group individuals with similar characteristics into clusters, and visualize these clusters in a 2D space without loss of information about any variable.

**Figure 5:**
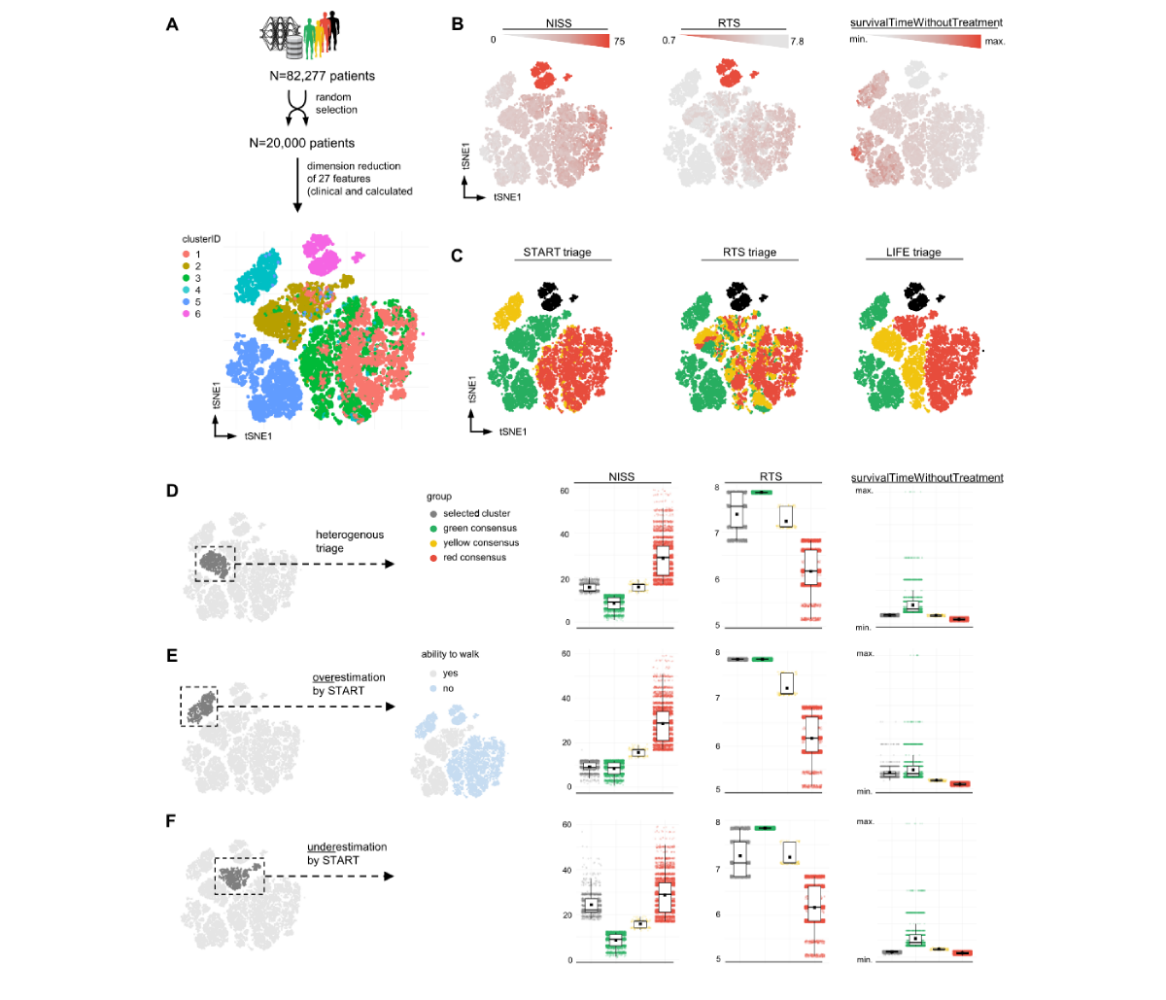
Multidimensional analysis of the patient database replicates triage clusters and identifies patients at risk for mistriage. (A) A total of 20,000 (24.3%) patients from the database were randomly selected. Next, 27 clinical and algorithm-calculated patient features were analyzed following a multidimensional mixed-data approach using the Gower distance and t-SNE (as specified in the Methods and Results sections). After dimensionality reduction, unsupervised clustering resulted in 6 specific patient clusters, which were subsequently analyzed. (B) Feature plots in t-SNE projection displaying the continuous distribution of LIFE triage–relevant parameters (NISS, RTS, and “survivalTimeWithoutTreatment”) over all patients analyzed. Cluster 6 can easily be identified as the “black” triage category. (C) Color-coded results of different triage algorithms for patients analyzed in the t-SNE projection. It can be clearly observed that some clusters or subclusters displayed different triage results. These clusters were analyzed in the next step. (D-F) Analysis of the highlighted clusters showing heterogeneous triage results (D), overestimation (E), and underestimation (F) using START. The variables NISS, RTS, and “survivalTimeWithoutTreatment” of the highlighted cluster were to all other patients, which all 3 triage algorithms consistently diagnosed as “green”/“yellow”/“red” (referred to as “consensus”). The LIFE triage model outperformed all other triage systems in terms of accordance with consensus triage. NISS: New Injury Severity Score; RTS: Revised Trauma Score; START: Simple Triage And Rapid Treatment; t-SNE: t-distributed stochastic neighbor embedding.

In this work, we used our artificial patient database and analyzed 27 clinical and algorithm-calculated patient features, as specified in Multimedia Appendix 1, Figure S7. The similarity and dissimilarity between patient features were calculated, as described in the Methods section, and the results are visualized as t-SNE plots in Figure 5A. Each point represents an individual patient, while the accumulation of points indicates patients with similar variables.

Feature plots were used to describe the distribution of the NISS, RTS, and “survivalTimeWithoutTreatment” over all analyzed patients (Figure 5B). We observed cluster 6 to have the highest NISS, the worst RTS, and the lowest “survivalTimeWithoutTreatment.” In conclusion, this cluster was represented by extremely critical patients, which would be set to the “black” triage category. We reviewed our hypothesis and plotted the triage results of the 3 different triage algorithms described previously (Figure 5C). Our hypothesis was confirmed by all triage algorithms. Strikingly, we found (sub)clusters of patients who were triaged heterogeneously (Figure 5D) or differently by the 3 triage algorithms (Figures 5E and 5F).

We first looked at the subcluster of heterogeneously triaged patients (Figure 5D). Although START identified all the patients as minor, the LIFE triage model classified them as delayed patients and the RTS classified them in the “green,” “yellow,” or “red” triage category. To evaluate which triage category is most likely to be the correct one, we compared the NISS, RTS, and “survivalTimeWithoutTreatment” of the patients from the selected subcluster with all other patients, where all 3 triaged algorithms consistently assigned patients to the “green,” “yellow,” or “red” triage category (ie, “consensus”). Surprisingly, when comparing the averages and distribution of individuals in the bar plots, the LIFE triage model seemed to outperform the other triage algorithms, as the patients from this subcluster were mostly relatable to patients in the “yellow” triage category, as identified only by our model.

There was also a cluster of patients that START assigned to the “yellow” triage category, while the RTS and LIFE triage models classified them in the “green” triage category. When comparing the NISS and RTS values of patients from these subclusters, it became clear that these patients were overtriaged by START and correctly triaged by the other algorithms. We investigated this issue and found the analyzed cluster to exclusively contain patients with good vital signs but unable to walk due to a minor injury of the lower extremity, marginally reduced vigilance, or low blood pressure with otherwise inconspicuous vital parameters. We concluded that categorization based on the ability to walk is a critical risk factor for mistriage in START. We further identified a subcluster of presumable underestimations by START (Figure 5F). In all depicted variables, patients from this cluster most likely resembled those in the “red” triage category, but START classified them in the “green” triage category. This patient subset included those with good overall vital parameters but poor prognosis due to high injury severity. Our proposed LIFE triage algorithm again successfully identified these patients as high priority and correctly assigned them to the category of immediate need for care.

## Discussion

### Principal Findings

In this study, we presented a novel approach for triage that no longer classifies patients into triage categories but ranks their urgency according to the anticipated survival time without intervention. This estimation was performed with a mathematical model evaluating current vital signs and injury severity. Our model successfully prognosticates the decline of the LIFE percentage and corresponding vital parameters and can thus evaluate the current and future urgency for treatment of a patient. To the best of our knowledge, this is one of the first studies to use a mathematical approach to examine mass casualty incident triage [9,27,28]. It is unique in terms of using triage ranking rather than triage categorization and in terms of implementing both vital parameters and injury severity using well-established scoring systems.

Triage is considered the cornerstone of effective disaster management and has significant implications for the overall medical response to critical incidents. However, for decades, triage algorithms have only marginally changed and have not kept pace with the technological advances of the current time. Established triage algorithms have been extensively examined, adapted, and validated in clinical use, yet their critical limitations have never been solved. Our proposed LIFE model tackles these limitations and sheds a novel light on the idea behind triage.

Currently, triage results in 4-5 categories of patients, who should be treated with different priorities. This is simple, fast, and effective but does not consider the variability of patients within a category and is consequently highly susceptible to mistriage. In contrast, our LIFE model ranks patients and maintains their individual variability. Triage categories can still be applied, but urgency ranking has the decisive advantage of precisely defining the specific patient who must be treated first.

Another critical factor is resource limitations. In such a case, triage should give higher priority to patients in less critical conditions but with higher chances of survival, thereby achieving an improvement in the expected number of lives saved [9]. Only a few algorithms consider triage in situations in which available health care resources are insufficient for the number and severity of casualties. However, those resource-sensitive triage algorithms leave the subjective decision of the “likelihood to survive with the given current resources” to health care professionals [29]. Our model indicates that the patient with the highest priority is automatically the patient with the lowest survival likelihood and can therefore clearly be identified. Furthermore, specific specialties or equipment can be proactively transferred to these patients.

Finally, current triage is a snapshot of momentary conditions and does not consider the future course. Patients with current good vital signs but severe injuries could mistakenly receive delayed treatment, while patients with worse but stable vital parameters could be prioritized. For that reason, the novel LIFE algorithm implements signs of both the current conditions (RTS) and the further progression of the patient (NISS). There is abundant evidence that the NISS can successfully predict the survival likelihood and outcome for patients with trauma [30-35], thereby underlining the validity of the use of this parameter in our model.

The proposed LIFE model offers numerous application possibilities. For prehospital usage, it could be used in mass casualty incidents for secondary or even primary surveys. Since our model is based on common parameters that are collected anyway during medical assessment, no delay is expected compared to current triage algorithms. We even expect our model to accelerate the overall medical response, as priority ranking could decisively help in treatment prioritization and reduce the time for decision-making.

Another field of application is simulations. The integration in either real-life simulations or computer-based simulations may allow supervisors of fictive scenarios to keep track of a large number of victims and check whether participants correctly prioritize the patients.

Furthermore, since our method introduces time as a critical variable for triage, all kinds of time-sensitive components of medical response can be investigated. As an example, the model could be used to determine which technical or medical resource is needed at which time point or which patients must be prioritized for transfer to hospital structures in the case of resource limitations.

### Limitations

However, there are a few limitations to this study. First, the mathematical model described here represents a proof-of-concept on a novel form of triage. Although implementing well-established, extensively validated, and tested trauma scores, the model itself has currently only been internally validated with a large-scale patient database.

Second, at its current stage, the model does not refer to concrete survival times, as these will need to be interpolated from real patient data in future studies. Concrete time frames are particularly important to anticipate the amount of resources needed in mass casualty incidents.

Third, the model implements the parameters from widely used trauma scores. However, some parameters might be more important than others, or relevant parameters could be missing. Two recent studies by Khorram-Manesh et al [36,37] have evaluated the most commonly used preexisting prehospital triage systems to create 1 universal translational triage tool. Interestingly, the Delphi-controlled meta-study identified algorithm parameters that are quite like those used in START triage and as also implemented by our proposed LIFE triage model. However, future application in real-life scenarios will be needed to evaluate the meaningfulness and significance of the model and its parameters. As new data become available and the nature of mass casualty events continues to evolve, it will be important to continually evaluate and improve the model. This could involve the use of machine learning and other advanced techniques to analyze large data sets and identify patterns and trends that could inform further refinements to the algorithm.

### Future Perspectives

Although the triage model has shown promising initial testing, the road ahead is still long, and further validation and optimization need to be done. One promising avenue for future application is the development of a smartphone-based app that incorporates the triage algorithm. The app could enable first responders to triage patients quickly and accurately in real time and communicate the triage results along with specific geolocations to incident commanders. Here, the algorithm could significantly help keep track of all casualties. As patients are continuously ranked by the algorithm, rescue resources can be transparently allocated according to patient priority and resource availability. Especially in large-scale mass casualty incidents, this approach could provide a more comprehensive picture of the situation on the ground and ultimately save more lives in disasters.

### Conclusion

In summary, we suggested an innovative approach to triage patients involved in disaster or mass casualty incidents, which no longer classifies patients into classic triage categories but prioritizes them according to a mathematical model, considering their dynamic vital parameters and injury severity. This triage strategy successfully tackles the limitations of current triage, implementing resource, time, and survival sensitivity. Hence, the model could substantially improve the medical response to disasters and ease the pressure on health care professionals who risk their lives to save others.

## Appendix

Multimedia Appendix 1 Supplementary materials.

## Abbreviations

AIS: Abbreviated Injury Scale
NISS: New Injury Severity Score
RTS: Revised Trauma Score
START: Simple Treatment And Rapid Triage
t-SNE: t-distributed stochastic neighbor embedding

## Data Availability

The artificial patient database generated and used in this study is publicly available online [38].
