# Supplementary material for: Development of an Anticipatory Triage-Ranking Algorithm Using Dynamic Simulation of the Expected Time Course of Patients With Trauma: Modeling and Simulation Study
Source: J Med Internet Res. 2023 Jun 15;25:e44042. doi: 10.2196/44042 (PMC10337428; doi:10.2196/44042)
Supplement: Multimedia Appendix 1 [file jmir_v25i1e44042_app1.docx]

## Supplementary Online Material to

# Development of an Anticipatory Triage-Ranking Algorithm by Dynamic Simulation of the Expected Time Course of Patients with Trauma: Modeling and Simulation Study

Manuel Sigle^1,2^; Leon Berliner^1^; Erich Richter, MD^1,3^; Mart van Iersel^4^; Eleonora Gorgati, MSc^1^; Ives Hubloue^5^; Maximilian Bamberg, MD^1^; Christian Grasshoff, MD, PhD^1^; Peter Rosenberger, MD, PhD^1^ and Robert Wunderlich, MD, MSc^1,6^*

1University Department of Anesthesiology and Intensive Care Medicine, University Hospital Tübingen, Eberhard Karls University, Tübingen, Germany.

^2^University Department of Cardiology and Angiology, University Hospital Tübingen, Eberhard Karls University, Tübingen, Germany.

^3^University Department of Pediatrics and Adolescent Medicine, Ulm University Medical Center, Ulm, Germany.

^4^ISEE (Interactive Simulation Emergency Exercise) support limited company, Wemmel, Belgium.

^5^Research Group on Emergency and Disaster Medicine, Vrije Universiteit Brussels, 1050 Brussels, Belgium.

^6^German Society for Disaster Medicine (DGKM Deutsche Gesellschaft für Katastrophenmedizin), Kirchseeon, Germany.

*corresponding author:

Robert Wunderlich, MD, MSc

University Department of Anesthesiology and Intensive Care Medicine, University Hospital Tübingen, Hoppe-Seyler-Straße 3, 72076 Tübingen, Germany

Robert.Wunderlich@med.uni-tuebingen.de

+49 7071 29-86564

## Table of Contents

| Content | Page(s) |
| --- | --- |
| Supplementary Methods 1: Data and analysis | 3 |
| Supplementary Methods 2: Design of mathematical model behind LIFE triage | 4-7 |
| Supplementary Methods 3: Generation of artificial patient database | 8 |
| Supplementary Methods 4: Computational assignment of triage categories | 10 |
|  |  |
| Supplementary Figure S1: Influence of NISS and RTS variables on LIFE model | 6 |
| Supplementary Figure S2: Backward calculation of vital parameter ranges at any time of LIFE model | 7 |
| Supplementary Figure S3: Details for generation of artificial patient database (Part 1) | 8 |
| Supplementary Figure S4: Details for generation of artificial patient database (Part 2) | 9 |
| Supplementary Figure S5: Programmatic logic of computational triage assignment | 10 |
| Supplementary Figure S6: Detailed patient profiles from the simulation described for figure 3 | 11 |
| Supplementary Figure S7: Details on multidimensional analysis of patient database | 12 |
|  |  |
| Additional references | 13 |

## Supplementary Methods 1: Data and Analysis

Most of the analysis for the present study was performed in RStudio (RStudio, PBC, Boston, USA)[39] running R version 4.0.5. Basic graphs were plotted with GraphPad Prism 9.1.2 (GraphPad Software, La Jolla, California, USA). The final graphics and layout were assembled in PowerPoint Version 2206 (Microsoft Corporation, Redmond, Washington, USA).

The mathematical backbone of our model, including the Boltzmann function and the basic implementation of RTS and NISS are shown in figure 1B. The artificial patient database can be accessed online through the following link: 10.5281/zenodo.7134900. The work steps for the application of the START and RTS Triage algorithms to our patient cohort are shown in Supplementary figures 3A and B.

## Supplementary Methods 2: Design of mathematical model behind LIFE triage

As mathematical backbone we used the Boltzmann function (figure 1b), a formula widely used in the bioinformatics field to visualize biological processes of increase or decline[12]. We then integrated the variables NISS (New Injury Severity Score[11]) and RTS (Revised Trauma Score[10]) into the basic function and applied minor mathematical modifications (1).

(1)

$$f\left( x \right)=RTS+ \frac{(Bottom-RTS)}{1+{exp}^{(\frac{t_{0.5}*RTS-NISS*x}{slope})}}+\Delta$$

(t0.5: time of 50% LIFE drop, ∆: correction factor to let y intercept equal normalized RTS score)

We used the New Injury Severity Score (NISS) as a measure of the severity of the patients’ injuries. Compared to the original Injury Severity Score (ISS) published in 1974[40], the revision of the score by the NISS improved outcome and mortality prediction, misclassification rates, sensitivity and accuracy.[11, 30-32, 35] The difference between the two scoring systems is that the NISS considers the patient’s most severe injuries regardless of body regions, unlike the ISS. The NISS is defined as “the sum of the squares of the Abbreviated Injury Scale (AIS) scores of each of the patient’s three most severe injury, regardless of the body region in which they occur” (figure 1C, (2)).[11] For the generation of our patient database, we used body locations as shown in figure 1C.

$$NISS={{AIS}_{A}}^{2}+{{AIS}_{B}}^{2}+{{AIS}_{C}}^{2}$$

(2)

To mathematically implement the vital parameters into the LIFE triage model, we used the Revised Trauma Score[10] (RTS). The calculation is based on the sum, differently weighted, of the points indicated by Glasgow Coma Scale (GCS), Systolic Blood Pressure (SBP) and Respiratory Rate (RR), as shown in figure 1D and (3).

$$RTS=0\boldsymbol{.}9368*GCS+0.7326*SBP+0.2908*RR$$

(3)

The “survivalTimeWithoutTreatment” was computed as x intercept (4). The “compensationTime” was defined as 10% decline of the initial “LIFE percentage” (5). The latter in turn was determined as a percentage from the maximum RTS.

(4)

$$f\left( x \right)=0=0$$

$$f\left( x \right)=0.9* =f(0)*f(0)$$

(5)

The calculation of vital parameters at any time assumes that vital signs change over time, while the severity of the injury remains constant. Considering this assumption, RTS values can be calculated backwards from the initial values by using fractions as stated in (6)-(8). As these RTS values correspond to vital parameter ranges, the latter can be estimated by our calculation.

$$GCS\left( x \right)=\frac{f(x)}{f(0)}\cdot{GCS}_{0}$$

(6)

$$SBP\left( x \right)=\frac{f(x)}{f(0)}\cdot{SBP}_{0}$$

(7)

$$RR\left( x \right)=\frac{f(x)}{f(0)}\cdot{RR}_{0}$$

(8)

(GCS/SBP/RR_0_: initial RTS values for GCS/SBP/RR)

We observed the following:

The course of the functions describes a pathophysiological compensation and decompensation period, as well as a period of irreversible decline of life (Supplementary figure 1A). The slope of decline is strongly associated with the injury severity, as integrated into the formula by NISS (Supplementary figure 1B). While a low NISS shows a very flat slope, a small increase in the injury severity strongly influences the slope. Simultaneously, greater severity above the level of a polytrauma patient (NISS > 15) shows a minor impact on the time course. We highlighted this behavior by plotting derivate functions from the LIFE formula (Supplementary figure 1C), pointing out the described pattern for “survivalTimeWithoutTreatment” and “compensationTime” (Supplementary figure 1C, D).

Besides the influence of NISS on the slope of the LIFE curve, we tried to make the RTS affect the shape of the graph. Deterioration of RTS between initial assessment and hospital arrival has been shown to be an independent predictor of mortality after hospitalization[41]. In our model, patients with poor initial vital signs should show a reduced initial LIFE percentage, calculated as follows:

(9)

$$f\left( x \right)=0=0$$

These patients have less resources for compensation and should therefore have a shorter time of compensation. This is shown in Supplementary figure 1D, for different RTS values with constant NISS values. As before, we plotted derivate functions from the LIFE formula for the “survivalTimeWithoutTreatment” and “compensationTime” (Supplementary figure 1E).

**A**

**B**

**C**

**E**

**D**

**Supplementary Figure S1: Influence of NISS and RTS variables on LIFE model**

**A** General course of life percentage during severe trauma, as proposed by our LIFE model. **B, C** Influence of different injury severities (by NISS) on the curve shape. Additional functions can be derived from the LIFE function, as demonstrated by the disproportionate dependence of the “survivalTimeWithoutTreatment” on the severity of the injury (**C, left**) or compensationTime (**C, right**). **D, E** Influence of different vital parameters (by RTS) on survival. While “survivalTimeWithoutTreatment” displays a nearly linear correlation with RTS (**E, left**), compensationTime is overly influenced by vital parameters (**E, right**)

Another feature of our mathematical model is the possibility to simulate temporal changes of vital parameters in trauma patients. This is especially useful for simulation purposes as for anticipatory resource management or evaluation of patient transportation priorities. The calculation of the vital parameters is possible due to two features of our mathematical model: 1) We expect the NISS to be constant, as the injury severity – in contrast to vital signs – normally doesn’t change over time. 2) The RTS is implemented as score and not just as vital signs, which allows to model disproportionate changes in vital signs. As an example, a deterioration in respiration normally leads to an increased respiratory rate, but when the patient reaches respiratory exhaustion, the respiratory rate drops below normal. We plotted the time course of the RTS components and their underlying vital parameter ranges in Supplementary figure 2B.

Obviously, only vital sign intervals can be calculated backwards, as these correspond to a specific RTS item. We expected the vital signs would change in a similar way to the time course of the “LIFE percentage”, with a period of compensation, decompensation, and irreversibility. As the vital parameter graph can be considered as derivative from the “LIFE percentage” graph, the exact same behavior was observed (black line in Supplementary figure 2A in comparison to colored lines). This Figure displays the time course of a patient with initial RTS values of GCS = 4 points, SBP = 3 points and RR = 2 points.

**A**

**B**

**Supplementary Figure S2: Backward calculation of vital parameter ranges at any time of LIFE model**

**A** Time course of different initial RTS values (GCS = 4, SBP = 3 and RR = 2). **B** By backward calculation over the LIFE model, vital parameters at any time point can be modeled within the ranges defined by RTS.

## Supplementary Methods S3: Generation of the artificial patient database

The workflow for generating the patient database is shown in figure 2A. For the combination of “type of trauma” and “location” we used the variables shown in Supplementary figure 3A. Conditional vital signs were assigned to the basic vital signs by the switches displayed in Supplementary figure 3B. A sample patient profile is shown in Supplementary figure 3C.

Basic vital parameters were defined as sets, containing specific ranges of vital signs for patients with varying injury severity (Supplementary figure 4A). Individual values were randomly selected from the ranges with a frequency distribution shown in Supplementary figure 4B.

| **RR** [min^-1^] | Skin color |
| --- | --- |
| > 15 | normal |
| 6-9 | cyanotic |
| 1-5 | cyanotic |
| 0 | grey |

| **Body temperature** [°C] | Skin touch |
| --- | --- |
| > 39 | hot |
| 35-37 | normal |
| 32-35 | cold/pale |
| < 32 | cold |

| **SBP**  [mmHg] | Capillary Refill Time [s] | Pulse palpability (radial) |
| --- | --- | --- |
| > 100 | 1 | yes |
| 80-100 | 2 | yes |
| 60-80 | 3 | no |
| < 60 | 4-10 | no |

**Type of trauma**

**(n = 14)**

**Location**

**(n = 13)**

Dislocation

Sprain/Strain

Fracture

Amputation

Crush injury

Nerve injury

Blast injury

Burn injury

Cold injury

Contamination injury

Organ injury

Inhalation injury

Drowning/Submersion

Internistic disease

Head

Face

Neck

Chest

Abdomen

Pelvic ring

Ribs

Arm

Hand/Finger

Leg

Foot

Back

Spine

**B**

**A**

| GCS | 13 |
| --- | --- |
| RR | 34 |
| SBP | 81 |

| RTS | 7.1082 |
| --- | --- |

| HR | 150/min |
| --- | --- |
| Temperature | 35°C |
| Skin color | normal |
| Skin touch | cold |
| Cap. Refill Time | 2 sec. |
| Pulse Palpability | yes |

| Trauma 1 | Amputation |
| --- | --- |
| Body Region 1 | Leg |
| AIS_A_ | 5 |
| Trauma 2 | Fracture |
| Body Region 2 | Arm |
| AIS_B_ | 3 |

| NISS | 34 |
| --- | --- |

| **Patient Profile #432** |
| --- |

**C**


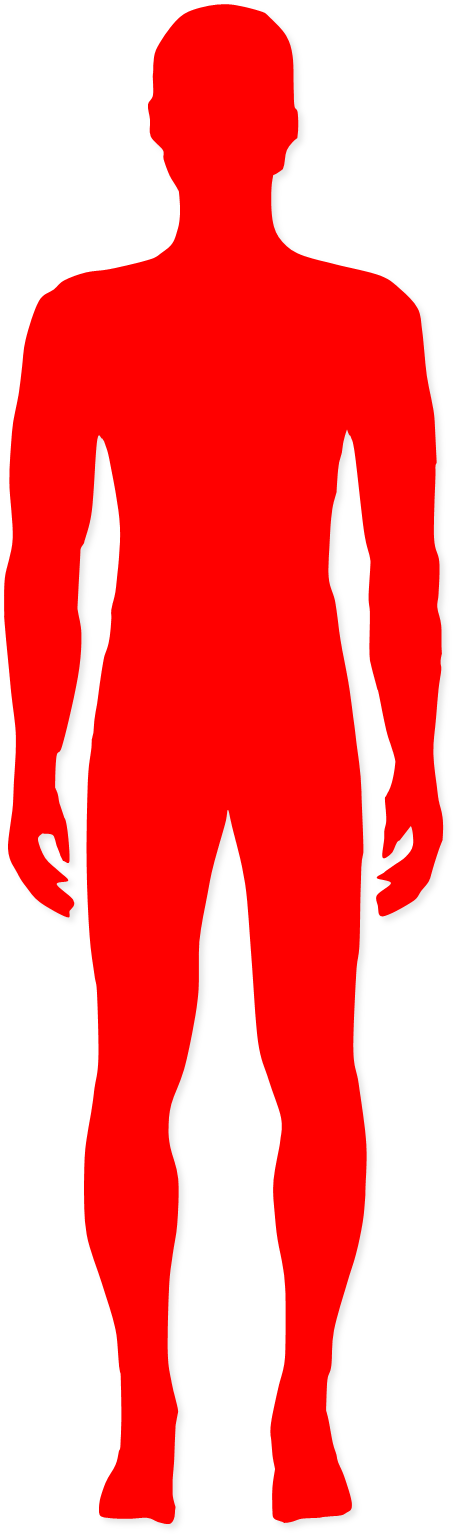

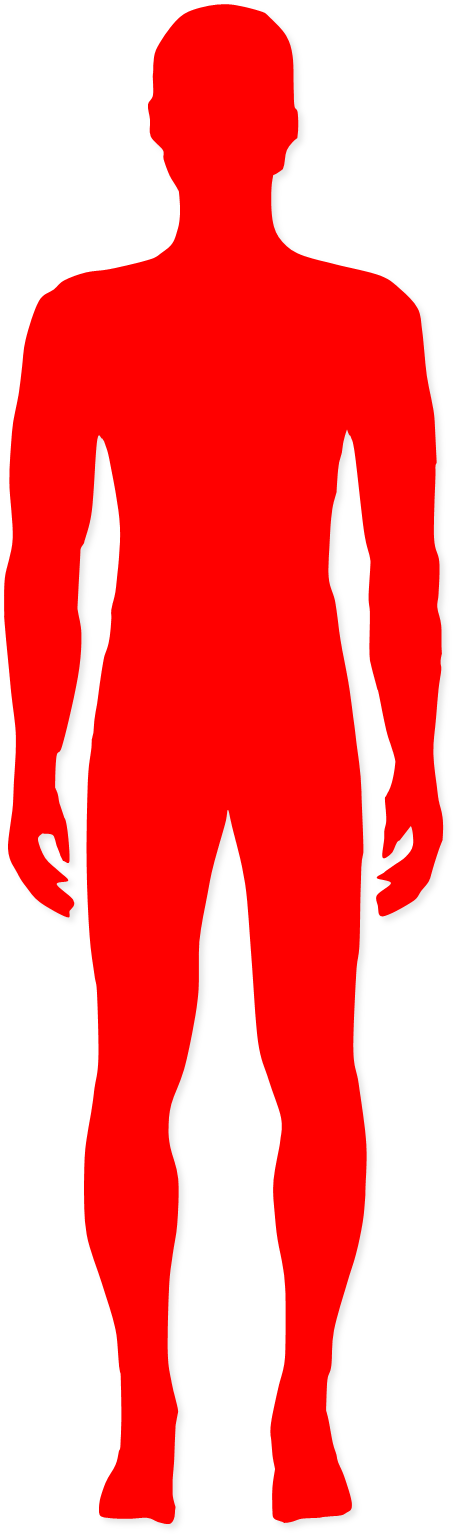


**Supplementary Figure S3: Details of the generation of the artificial patient database (Part 1)**

**A** Variables “type of trauma” and “location” used for generating the artificial patient database. **B** Depending variables were automatically assigned to basic vital signs defined by vital parameter sets (Supplementary figure 4A). **C** Example extracted from the artificial patient database (Patient #432) with a severe injury pattern.


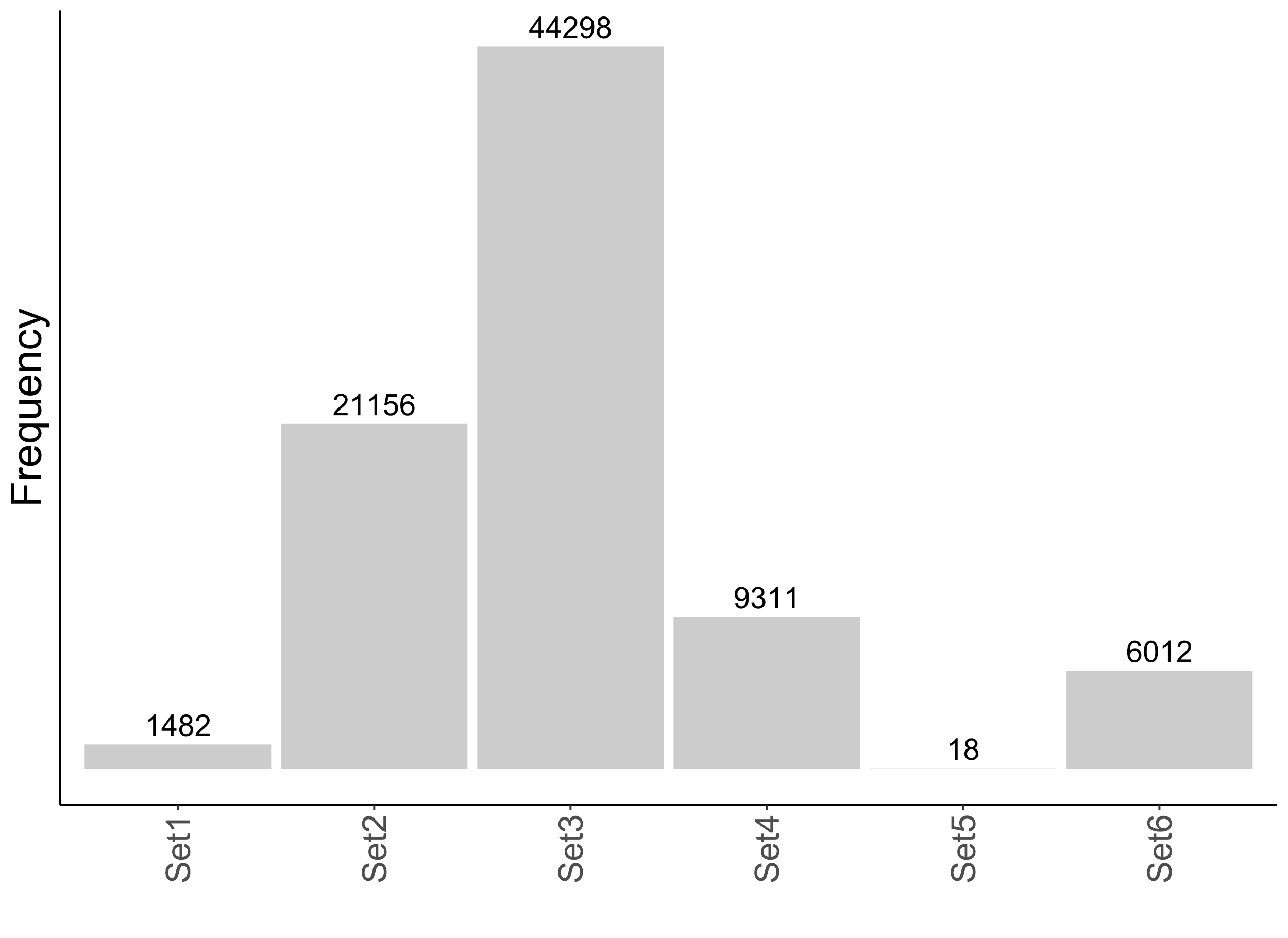


**A**

**B**

| **Set** | **Injury Severity** | **AIS [Points]** | **Description of vital signs** | **GCS [Points]** | **SBP [mmHg]** | **RR**  **[min^-1^]** | **Heart rate**  **[min^-1^]** | **Body temperature [°C]** |
| --- | --- | --- | --- | --- | --- | --- | --- | --- |
| 1 | minor | 1 | equals healthy | 15 | 110-130 | 12-15 | 50-90 | 36-38 |
| 2 | moderate | 2 | moderate ill | 15 | 100-200 | 15-20 | 90-120 | 36-39 |
| 3 | serious | 3 | shock | 12-14 | 80-100 | 20-40 | 120-200 | 35-37 |
| 4 | severe | 4 | severe shock | 10-13 | 70-90 | 20-40 | 140-200 | 34-36 |
| 5 | critical | 5 | decompens. | 4-9 | 40-80 | 3-10 | 20-40 | 33-35 |
| 6 | maximal | 6 | dead/reanim. | 3 | 0-40 | 0 | 0 | 28-34 |

**C**


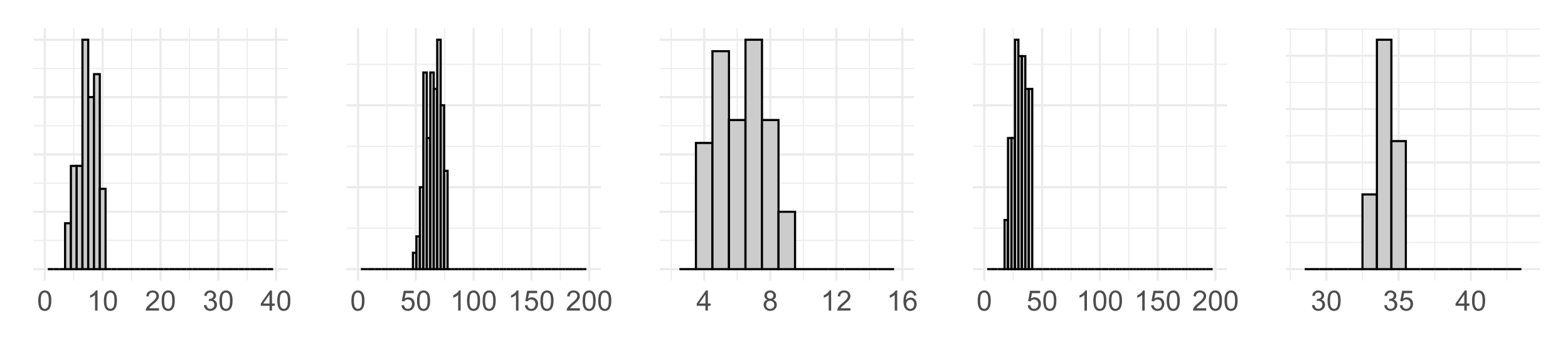

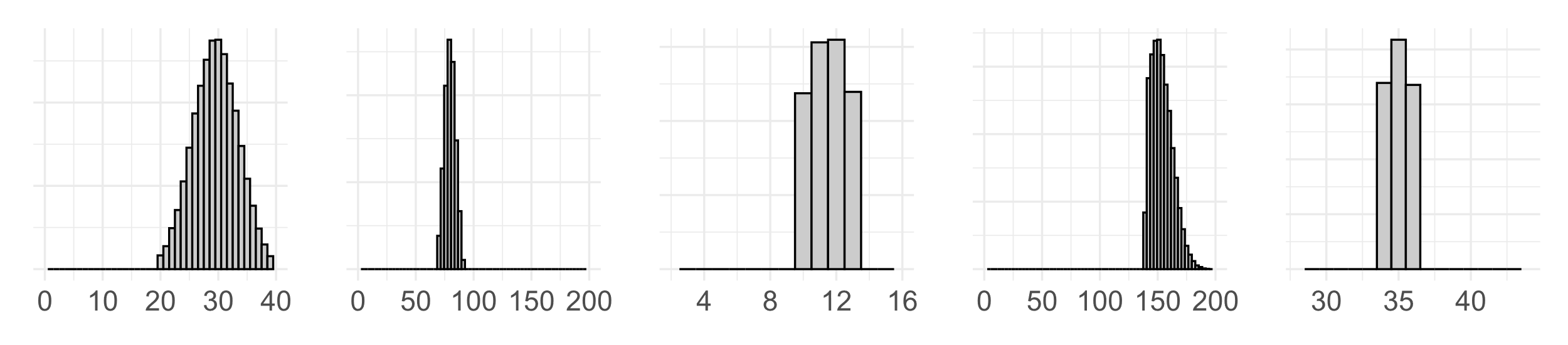

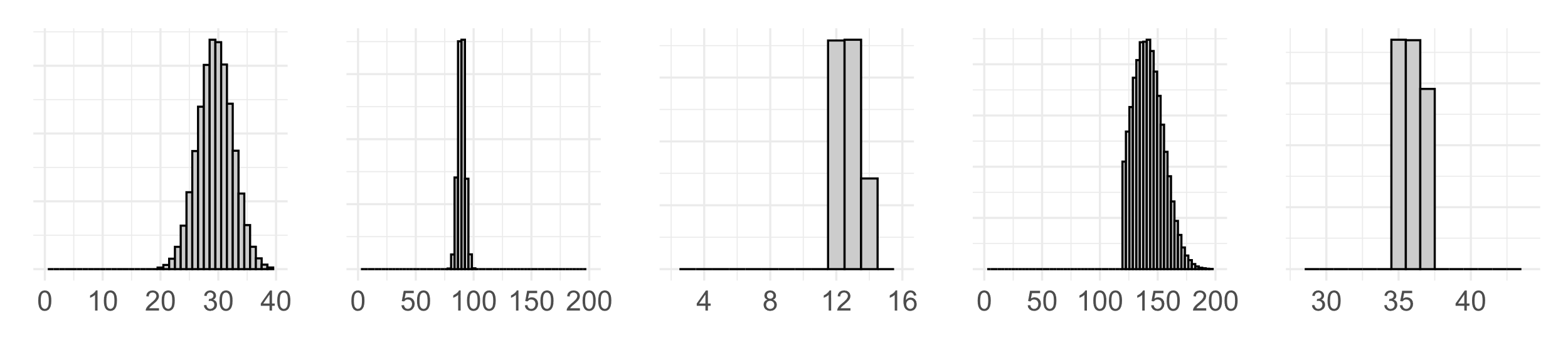

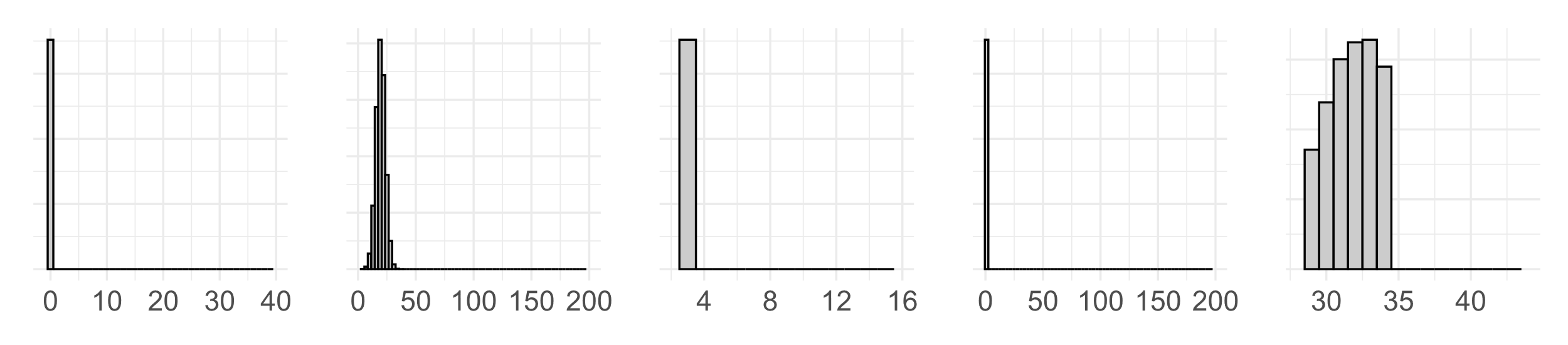

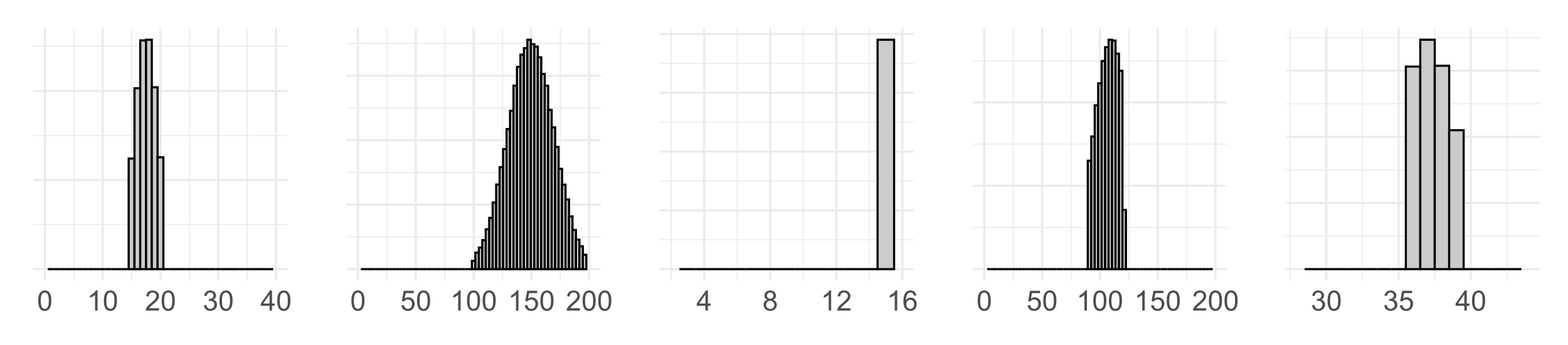

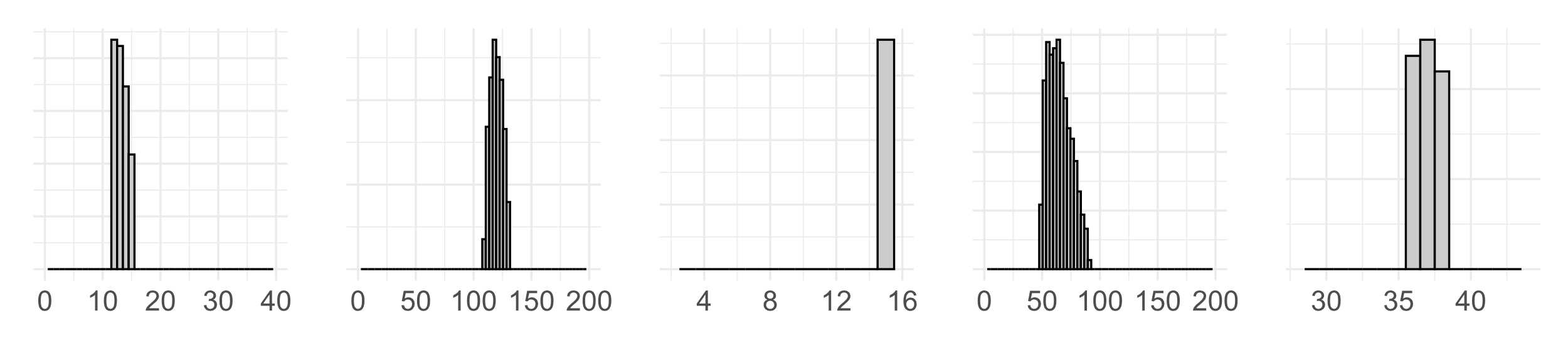


**RR SBP GCS Heart Rate Body Temperature**

**Set 1**

**Set 2**

**Set 3**

**Set 4**

**Set 5**

**Set 6**

**Supplementary Figure S4: Details for generation of artificial patient database (Part 2)**

**A** Vital signs sets with realistic ranges of variation. A specific set was assigned to each patient, depending on the patient’s injury severity. **B** The frequency distribution of the assigned vital signs sets is displayed as a histogram. **C** The frequency distribution of vital signs defined by ranges in the sets of **A**.

## Supplementary Methods 4: Computational assignment of triage categories

We used START Triage[42] and RTS Triage[24, 43] to compare the triage results with those of our LIFE model. The programmatic logic is shown in Supplementary figure 5.

**A**

**B**

# Triage based on RTS

RTSTriage <- function(RTS.GCS, RTS.SBP, RTS.RR) {

if (RTS.GCS+RTS.SBP+RTS.RR == 12) {

"GREEN"

} else if (RTS.GCS+RTS.SBP+RTS.RR == 11) {

„YELLOW"

} else if (RTS.GCS+RTS.SBP+RTS.RR >2 & RTS.GCS+RTS.SBP+RTS.RR <11) {

"RED"

} else {

„BLACK"

}

}

# Triage based on START Algorithm

STARTTriage <- function(walkAbility, RR, capillaryRefillTime, GCS) {

if (walkAbility == "yes") {

"GREEN"

} else if (RR<1) {

"BLACK"

} else if (RR>30) {

"RED"

} else if (capillaryRefillTime>2) {

"RED"

} else if (GCS<14) {

"RED"

} else {

"YELLOW"

}

}

**Supplementary Figure S5: Programmatic logic of computational triage assignment**

**A** Programmatic workflow of the application of START Triage on our artificial patient database. Instead of “patient obeys commands” we set up the full GCS for the evaluation of the patient’s neurology. **B** RTS Triage application programmatic workflow. Please note that for Triage, RTS values are simply summed up without different weights as originally used in RTS.

**A**

|  | kindOfTrauma  1 | bodyRegion  1 | AIS1 | kindOfTrauma  2 | bodyRegion  2 | AIS2 | kindOfTrauma  3 | bodyRegion  3 | AIS3 | NISS | GCS | RR | SBP | HR | BT | CRT | PP | SkinColor | SkinTouch | walkAbility | RTS.  GCS | RTS.  SBP | RTS.  RR | RTS | swt%max | ct%max | TRIAGE.  START | TRIAGE.  RTS | TRIAGE.  LIFE |
| --- | --- | --- | --- | --- | --- | --- | --- | --- | --- | --- | --- | --- | --- | --- | --- | --- | --- | --- | --- | --- | --- | --- | --- | --- | --- | --- | --- | --- | --- |
| #1 | Amputation | Arm | 4 | Amputation | Leg | 5 | Crush injury | Head | 5 | 66 | 8 | 7 | 59 | 40 | 33 | 4 | no | cyanotic | cold/pale | no | 2 | 2 | 2 | 3.9204 | 1.90445656 | 0.92390159 | RED | RED | BLACK |
| #2 | Crush injury | Hand/Finger | 1 | Crush injury | Head | 5 | Fracture | Arm | 3 | 35 | 13 | 33 | 81 | 142 | 35 | 2 | yes | normal | normal | yes | 4 | 3 | 3 | 6.8174 | 5.13061293 | 4.40150656 | GREEN | RED | RED |
| #3 | Dislocation | Arm | 2 | Dislocation | Leg | 2 | Fracture | Spine | 4 | 24 | 14 | 28 | 91 | 135 | 35 | 2 | yes | normal | normal | no | 4 | 4 | 4 | 7.8408 | 8.33333333 | 8.33333333 | YELLOW | GREEN | RED |
| #4 | Dislocation | Arm | 2 | Fracture | Head | 4 | NA | NA | NA | 20 | 13 | 30 | 90 | 141 | 36 | 2 | yes | normal | normal | yes | 4 | 4 | 3 | 7.55 | 9.70764252 | 9.32041522 | GREEN | YELLOW | RED |
| #5 | Crush injury | Arm | 2 | Dislocation | Leg | 2 | Fracture | Leg | 3 | 17 | 14 | 26 | 88 | 136 | 35 | 2 | yes | normal | normal | no | 4 | 3 | 4 | 7.1082 | 10.9017692 | 9.79696657 | YELLOW | YELLOW | YELLOW |
| #6 | Crush injury | Arm | 2 | Fracture | Leg | 3 | NA | NA | NA | 13 | 12 | 28 | 87 | 150 | 36 | 2 | yes | normal | normal | no | 3 | 3 | 4 | 6.1714 | 12.8426527 | 9.85683475 | RED | RED | YELLOW |
| #7 | Crush injury | Chest | 3 | Sprain/Strain | Neck | 2 | NA | NA | NA | 13 | 13 | 36 | 88 | 134 | 36 | 2 | yes | normal | normal | yes | 4 | 3 | 3 | 6.8174 | 13.8131887 | 11.85021 | GREEN | RED | YELLOW |
| #8 | Dislocation | Arm | 2 | Fracture | Ribs | 2 | NA | NA | NA | 8 | 15 | 17 | 146 | 109 | 36 | 1 | yes | normal | normal | yes | 4 | 4 | 4 | 7.8408 | 25 | 25 | GREEN | GREEN | GREEN |
| #9 | Dislocation | Foot | 2 | NA | NA | NA | NA | NA | NA | 4 | 15 | 15 | 132 | 116 | 37 | 1 | yes | normal | normal | no | 4 | 4 | 4 | 7.8408 | 50 | 50 | YELLOW | GREEN | GREEN |
| #10 | Dislocation | Hand/Finger | 1 | Sprain/Strain | Foot | 1 | NA | NA | NA | 2 | 15 | 14 | 116 | 74 | 37 | 1 | yes | normal | normal | yes | 4 | 4 | 4 | 7.8408 | 100 | 100 | GREEN | GREEN | GREEN |

GCS: Glasgow Coma Scale

RR: Respiratory Rate

SBP: Systolic Blood Pressure

HR: Heart Rate

BT: Body Temperature

CRT: capillary refill time

PP: pulse palpability

RTS.GCS/SBP/RR: RTS Points assigned to vital signs

swt%max: % survivalTimeWithoutTreatment of maximal value (scenario specific)

ct%max: % compensationTime of maximal value (scenario specific)

**Supplementary Figure S6:**  **Detailed patient profiles from the simulation described for figure 3.**


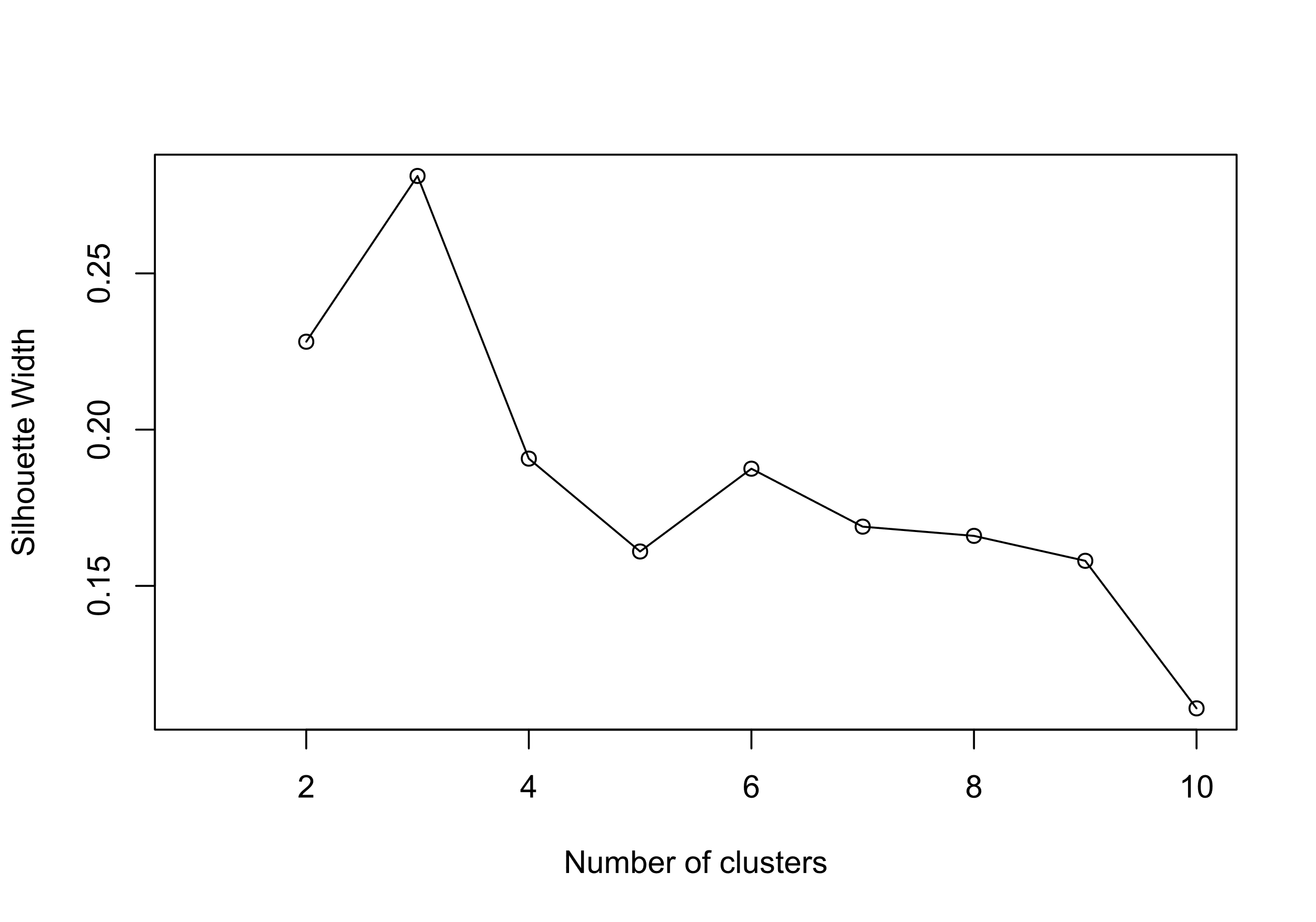


k = 6

**A**

**B**

| **Variable** | **Weight** | **Scale** |
| --- | --- | --- |
| traumaType1 | 1 | N |
| location1 | 1 | N |
| AIS1 | 1 | O |
| traumaType2 | 1 | N |
| location2 | 1 | N |
| AIS2 | 1 | O |
| traumaType3 | 1 | N |
| location3 | 1 | N |
| AIS3 | 1 | O |
| NISS | 1 | I |
| GCS | 1 | I |
| RR | 1 | I |
| SBP | 1 | I |
| HR | 1 | I |
| BT | 1 | I |
| CapillaryRefillTime | 1 | I |
| PulsePalpability | 1 | N |
| SkinColor | 1 | N |
| SkinTouch | 1 | N |
| walkAbility | 1 | N |
| RTS | 1 | I |
| LIFE.t0 | 1 | I |
| severityFactor | 1 | I |
| survivalTimeWithoutTreatment | 1 | I |
| TRIAGE.START | 1 | N |
| TRIAGE.RTS | 1 | N |
| TRIAGE.LIFE | 1 | N |

**Supplementary Figure S7: Details on the multidimensional analysis of patient database**

**A** Variables used for the multidimensional analysis. Gower distance was calculated with similar weights of variables. Scale N = nominal, O = ordinal, I = interval. **B** Silhouette width plot to evaluate the correct cluster size. We decided to use k = 6 clusters to avoid the underrepresentation of small patient cohorts.

## Additional references

39. RStudio-Team. RStudio: Integrated Development for R.: http://www.rstudio.com/; 2020; Available from: http://www.rstudio.com/.

40. Baker SP, OʼNeill B, Haddon W, Long WB. The Injury Severity Score. The Journal of

41. Lichtveld RA, Spijkers AT, Hoogendoorn JM, Panhuizen IF, van der Werken C. Triage Revised Trauma Score change between first assessment and arrival at the hospital to predict mortality. Int J Emerg Med. 2008 Apr;1(1):21-6. PMID: 19384497. doi: 10.1007/s12245-008-0013-7.

42. Newport Beach Fire and Marine Department, Beach HHiN. START Adult Triage Algorithm. Radiation Emergency Medical Management: REMM (US Department of Health and Human Services). 1983.
